# Research on blasting cumulative dynamic damage of surrounding rock in step construction tunnel

**DOI:** 10.1038/s41598-023-28900-w

**Published:** 2023-02-03

**Authors:** Yaozhong Cui, Bo Wu, Guowang Meng, Shixiang Xu

**Affiliations:** 1 College of Civil Engineering and Architecture, Guangxi University, Nanning, 530004 Guangxi China; 2 School of Civil and Architectural Engineering, East China University of Technology, Nanchang, 330013 Jiangxi China

**Keywords:** Civil engineering, Computational methods

## Abstract

In the process of cyclic blasting during tunnel excavation, the reserved surrounding rock sustains irreparable damage accumulation. For safe tunnel construction, it is imperative to understand the characteristics of blasting dynamic cumulative rock damage. Sonic wave test and numerical simulation methods were applied to the research. The JH-2 model was adopted as the damage model of surrounding rock. Based on the data transfer method between solvers in ABAQUS software, the cumulative damage was calculated. The damage characteristics were obtained by combining the sonic wave test results. According to the research findings, the entire reserved surrounding rock has periodic damage characteristics. Each periodic damage area has a funnel shape along the tunnel’s longitudinal direction, with a length of 160 cm, and 1.07 times the excavation footage. The latter excavation footage's blasting effect on the damaged area of the previous footage rock is 40 cm long, with three cumulative damage patterns. The three cumulative damage patterns more clearly reveal the surrounding rock's additional damage law, the degree of additional damage is greatest with the distance of 5–20 cm from the latter excavation footage. The research can provide appropriate theoretical guidance for the design of the step-blasting construction tunnel's blasting scheme and lining.

## Introduction

Blasting excavation is widely used in tunnel engineering because of its high efficiency and economic advantages. When the energy from an explosive explosion breaks and throws the excavated rock mass, it will inevitably damage the reserved surrounding rock, reducing its integrity and degrading its mechanical properties, affecting tunnel construction safety. Moreover, additional blasting loads will be applied to the reserved surrounding rock, causing continual damage accumulation. Therefore, studying the features of blasting cumulative dynamic damage of surrounding rock is crucial for the safe construction of drilling and blasting tunnels.

The rock blasting damage model has been studied extensively by academics. Langefors^1^ believes blasting damage is created by explosion stress wave propagation, reflection, and contact. It stimulates and extends rock fissures, lowering their mechanical characteristics. Based on this understanding, relevant researchers developed three blasting damage models: the GK model^2^, TCK model^3^, and KUS model^4^. HAMDI^5^ and LI^6^ utilized the three blasting damage models and numerical simulation methods to study the damage evolution, measurement, and evaluation of rock mass under blasting. According to continuum damage mechanics and critical tensile strain criterion, YANG^7^ and LIU^8^ proposed a blasting damage model that can comprehensively reflect the correlation among damage variables, crack density, and strain rate.

Furthermore, rock mass exists in the initial in-situ stress coupled with gravity stress and tectonic stress, which has a significant influence on the propagation of explosive stress waves, the development of rock cracks, and the damage to surrounding rock., rock mass exists in the initial in-situ stress coupled with gravity stress and tectonic stress, which has an significant influence on the propagation of explosive stress waves, and surrounding rock damage. Therefore, in-situ stress cannot be disregarded when studying the damage caused by blasting, and many academics have studied it. Through experimental research, HE^9^ and ZHANG^10^ found that cracks under blasting load often propagate along the direction of principal stress. In addition, the study of HE^9^ showed that the rock fracture zone would decrease with the increase of compressive stress. XIE^11^ and YI^12^ studied the blasting damage of rock mass under in-situ stress by numerical simulation and obtained the same crack development rule as above. TAO^13^ simulated single-hole blasting of rock mass under in-situ stress and found that it could reduce cracking. In a specific tunneling project, RAMULU^14^ used extensometer and pinhole camera tests to investigate cyclic blasting's influence on rock damage. The research found that rock damage near the blasting area was caused by high-frequency vibration, while rock damage far away from the blasting hole was caused by low-frequency vibration. LUO^15^ carried out a numerical simulation on the blasting excavation of a diversion tunnel and compared the results with the field-measured values. It was found that the blasting vibration velocity and surrounding rock deformation were closer to the measured values after considering the accumulated damage of surrounding rock. The cumulative damage effect of surrounding rock should be considered in the numerical simulation.

The above studies indicate that the appropriate rock damage model should be selected, and the effect of in-situ stress should be considered to explore the cumulative damage of reserved surrounding rock. However, the research status focuses on the damage theory of rock under blasting load, on-site monitoring, and safety evaluation. There are few studies on the cumulative dynamic damage of reserved surrounding rock in the step-method cyclic blasting construction. The JH-2 rock model built in ABAQUS software was used to conduct a precise numerical simulation of cyclic blasting excavation of a tunnel. The calculation results were compared with the field sonic test results to study the cumulative damage characteristics of the reserved surrounding rock.

## Project overview

### Engineering background

**Figure 1:**
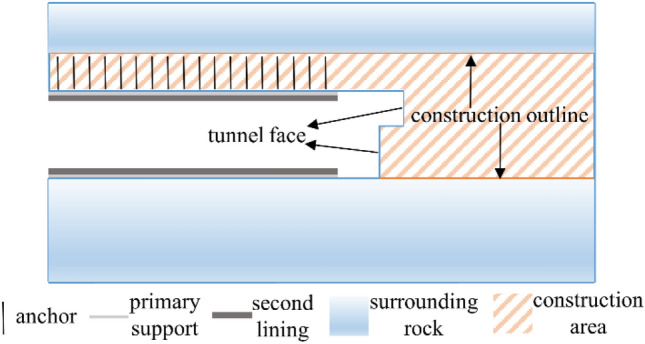
The tunnel construction diagram.

**Figure 2:**
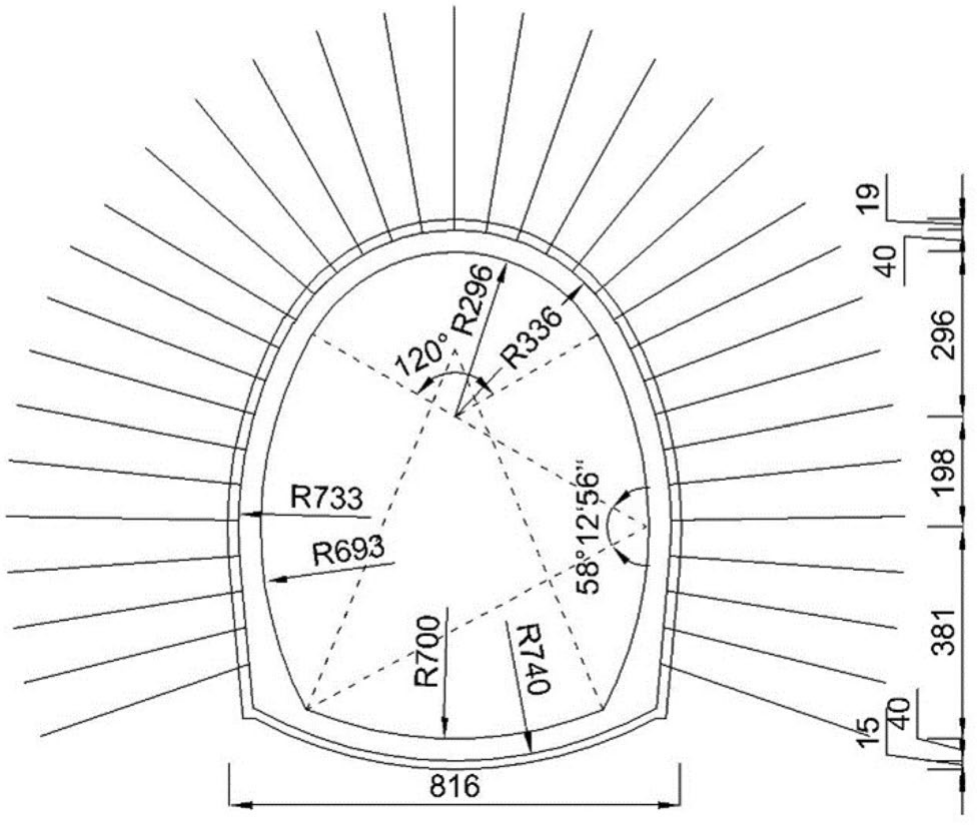
The tunnel dimension plot (cm).

Jinjing Tunnel is located in Sanming City, Fujian Province, China. It is a single-track tunnel of 7292 m in length. It passes through the surrounding rocks of class II, III, IV, and V, and the length for the surrounding rock of class II is 1540 m. To lessen the vibration produced by blasting to the nearby structures, a step-by-step temporary inverted arch construction method was chosen for the shallowly buried part of class II. The tunnel construction diagram is shown in Fig. 1, and the tunnel dimension plot is shown in Fig. 2.

### Blasting scheme

**Figure 3:**
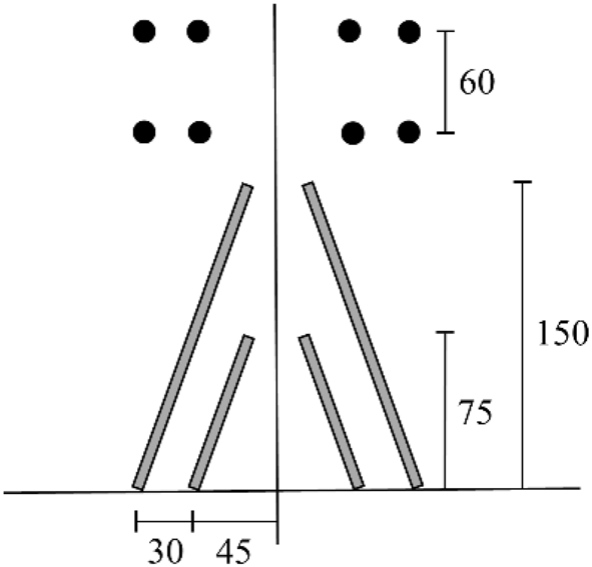
Compound wedge-shaped cutting hole layout scheme (cm).

The construction was done in accordance with the “short excavation, weak blasting” theory, and the micro-difference, division, multi-segment, and multiple weak blasting techniques were used. The temporary invert method of lower and down steps was used for construction, with a single cyclic excavation of 1.5 m. The cutting hole is compound wedge-shaped, and the layout is shown in Fig. 3. In Fig. 3, the upper part is the front view, which shows the arrangement of the cutting holes on the tunnel face. The bottom face is the top view, which shows the position and length of the cutting holes in the rock.

### Sonic wave test of the surrounding rock

**Figure 4:**
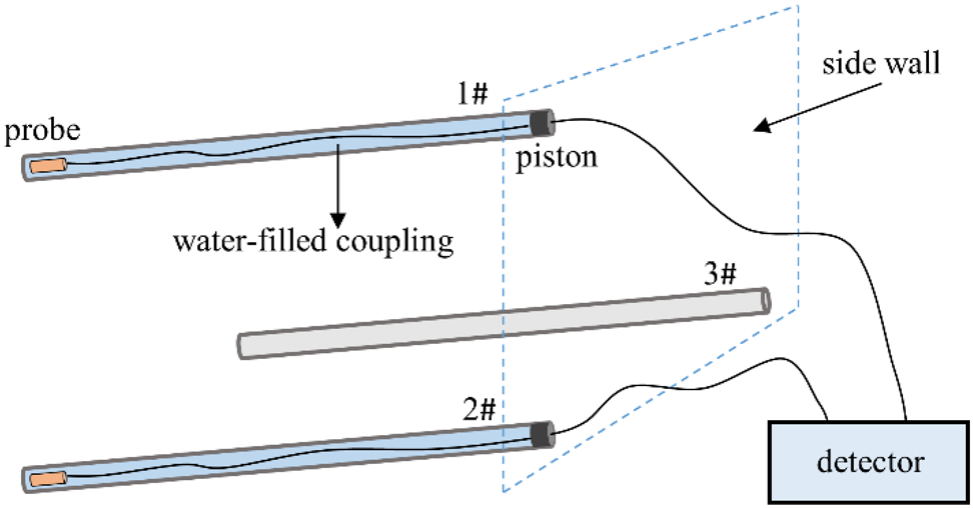
Two-hole sonic wave method test plan.

Changes in sonic wave velocity in a rock mass can better reflect the degree and range of rock mass damage following blasting and give data for studies from construction sites^16^. The RSM-SY6 detector is used to carry out sonic testing on the surrounding rock, and the test scheme is shown in Fig. 4. The test holes are arranged at the flat part of the arch waist. The depth of the test holes is 4 m, and the test holes are tilted downward and at a 5° Angle with the horizontal plane. The test holes are parallel, and the adjacent spacing is 60 cm. There was no rock debris in the test chamber during the test, and the cavity was coupled with water. The sonic probe was moved outward by 20 cm for each test, and 21 tests were performed in each group.

**Figure 5:**
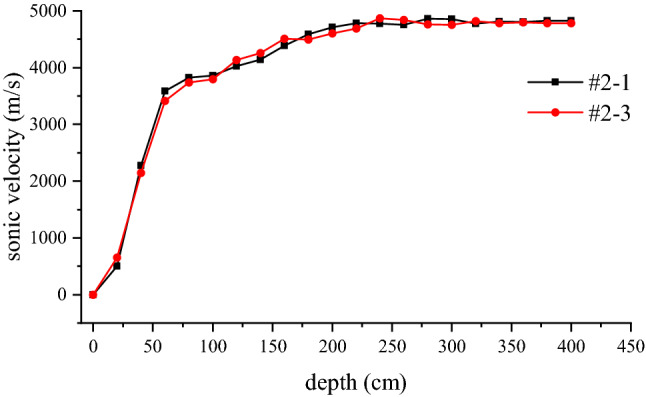
Sonic velocity-depth curves of surrounding rock.

In order to obtain the longitudinal wave velocities of damaged and intact surrounding rock, the sonic wave velocity of excavated rock mass is tested.The curve of the wave velocity varying along the depth of the test hole is shown in Fig. 5. In Fig. 5, #2–1 represents the rock sonic wave velocity between hole 2# and hole 1#, #2–3 represents the rock sonic wave velocity between hole 2# and hole 3#. Due to water seepage at the hole opening, the wave velocity data at the hole opening are incomplete. The wave velocity curve of the reserved surrounding rock shows that the wave velocity varies significantly in the depth range of 0.4–2.5 m, with the wave velocity rising from 2145 to 4845 m·s^−1^. When the depth is greater than 2.5 m, the variation of sonic wave velocity tends to be stable. Judging from the variation trend of the wave velocity, The wave velocity of the undamaged surrounding rock can be taken as the average wave velocity of 4817 m·s^−1^ at a depth of 2.5–4 m of the test hole.

## Rock damage model

Since the blasting load is merely a type of force, how the surrounding rock reacts to it depends on its mechanical characteristics and damage model. As a result, the damage model of the surrounding rock is crucial to the analysis of this work.

In the numerical simulation, HJC model^17^, JH-1 model^18^, and JH-2 model^19^ are often used to describe the dynamic mechanical behavior of brittle materials under considerable strain, high strain rate, and high-pressure loads. The JH-2 model is an improvement over the JH-1 model. Pressure-dependent strength, damage, fracture, bulking, strain rate effects, and significant strength after fracture are all taken into account in the JH-2 model^20^. The model consists of strength, damage, and pressure. The strength, damage, and pressure state equations of the JH-2 model are described below^21,22^.

### Strength of model

**Figure 6:**
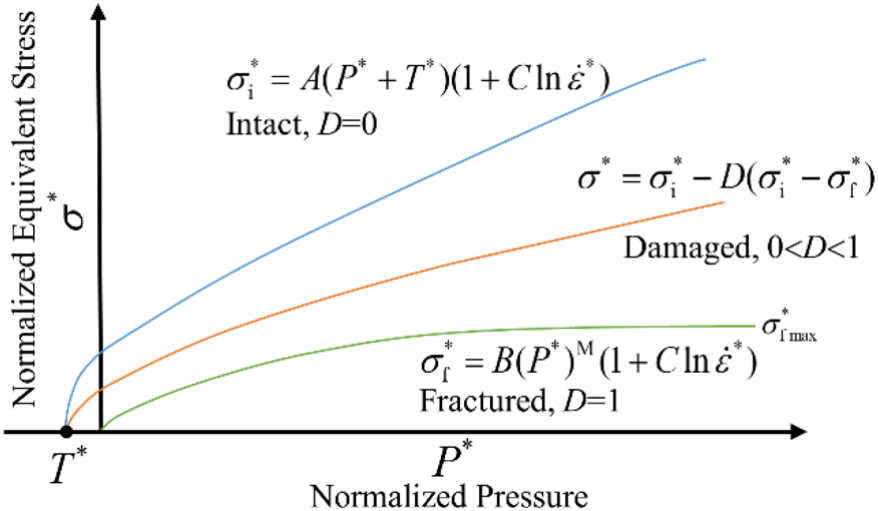
Strength model of the JH-2 constitutive model.

The strength curve of the continuum model of JH-2 is shown in Fig. 6, where three different curves describe three states of materials: the intact state, the damaged state, and the fractured state. The state of the material has its corresponding strength equation, which represents the relationship between normalized equivalent stress and normalized pressure. The equation of normalized equivalent stress is as follows:

1 $$\sigma^{*} = \sigma_{\text{i}}^{*} - D(\sigma_{\text{i}}^{*} - \sigma_{{\rm f}}^{*} ) = \frac{\sigma }{{\sigma_{{{\rm HEL}}} }}$$

where $\sigma_{\text{i}}^{*}$ is the normalized intact equivalent stress; $\sigma_{{\rm f}}^{*}$ is the normalized fracture stress; *D* is the damage factor(0 < *D* < 1); *σ* is the actual equivalent stress; $\sigma_{{{\rm HEL}}}$ is the equivalent stress in the Hugoniot elastic limit state, It represents the net compressive stress including hydrostatic pressure and deviator stress components^19^.

0 < *D* < 1, the material is in the damaged state and the normalized equivalent stress $\sigma^{*}$ is calculated by formula (1), corresponding to the orange curve in Fig. 6. *D* = 0, the material is in the intact state. Substituting *D* = 0 into formula (1), $\sigma^{*} { = }\sigma_{\text{i}}^{*}$ is obtained, and $\sigma_{\text{i}}^{*}$ is calculated by formula (3), which corresponds to the blue curve in Fig. 6. *D* = 1, the material is in the fractured state. Substituting *D* = 1 into formula (1), $\sigma^{*} { = }\sigma_{{\rm f}}^{*}$ is obtained, and $\sigma_{\rm {f}}^{*}$ is calculated by formula (4), which corresponds to the green curve in Fig. 6.

The calculation formula of *σ* is as follows:

2 $$\sigma = \sqrt {\frac{1}{2}\left[ {\left( {\sigma_{1} - \sigma_{2} } \right)^{2} + \left( {\sigma_{1} - \sigma_{3} } \right)^{2} + \left( {\sigma_{2} - \sigma_{3} } \right)^{2} } \right]}$$

where $\sigma_{1}$ and $\sigma_{2}$ are $\sigma_{3}$ three principal stresses.

The calculation formula of $\sigma_{i}^{*}$ and $\sigma_{f}^{*}$ is as follows:

3 $$\sigma_{i}^{*} = A(P^{*} + T^{*} )^{N} (1 + C\ln \dot{\varepsilon }^{*} ) \le \sigma_{i}^{\max }$$

4 $$\sigma_{f}^{*} = B(P^{*} )^{M} (1 + C\ln \dot{\varepsilon }^{*} ) \le \sigma_{f}^{\max }$$

where *A*, *B*, *C*, *M*, and *N* are material parameters; $\sigma_{i}^{\max }$ is the limit value of $\sigma_{i}^{*}$; $P^{*}$ is the normalized pressure, the calculation formula of $P^{*}$ is as follows:

5 $$P^{*} = \frac{P}{{P_{HEL} }}$$

where P is the actual hydrostatic pressure; $P_{HEL}$ is the hydrostatic pressure in the elastic limit state of Hugoniot.

$T^{*}$ is the normalized maximum tensile hydrostatic pressure, and its calculation formula is as follows:

6 $$T^{*} = \frac{T}{{T_{HEL} }}$$

where *T* is the maximum tensile hydrostatic pressure of the material and $T_{HEL}$ is the tensile hydrostatic pressure in the elastic limit state of Hugoniot.

$\dot{\varepsilon }^{*}$ is the dimensionless strain rate, which is calculated as follows:

7 $$\dot{\varepsilon }^{*} = \frac{{\dot{\varepsilon }}}{{\dot{\varepsilon }_{0} }}$$

where $\dot{\varepsilon }$ is the actual equivalent strain rate, $\dot{\varepsilon }_{0}$ is the reference strain rate, and takes the value of 1 s^−1^.

The calculation formula of $\dot{\varepsilon }$ is as follows:

8 $$\dot{\varepsilon } = \sqrt {\frac{2}{9}\left[ {\left( {\dot{\varepsilon }_{x} - \dot{\varepsilon }_{y} } \right)^{2} + (\dot{\varepsilon }_{x} - \dot{\varepsilon }_{z} )^{2} + \left( {\dot{\varepsilon }_{y} - \dot{\varepsilon }_{z} } \right)^{2} + \frac{3}{2}\left( {\dot{\gamma }_{xy}^{2} + \dot{\gamma }_{xz}^{2} + \dot{\gamma }_{yz}^{2} } \right)} \right]}$$

where $\dot{\varepsilon }_{x}$,$\dot{\varepsilon }_{y}$ and $\dot{\varepsilon }_{z}$ are three principal strain rates; $\dot{\gamma }_{xy}^{2}$,$\dot{\gamma }_{xz}^{2}$ and $\dot{\gamma }_{yz}^{2}$ are three shear strain rates.

### Damage of model

**Figure 7:**
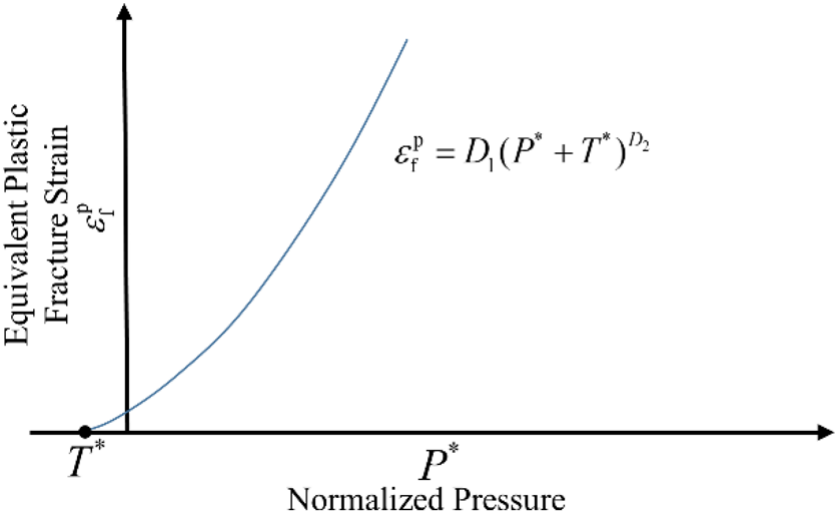
Damage model of the JH-2 constitutive model.

The damage curve of the JH-2 continuum model is shown in Fig. 7. From Fig. 7, the material's damage increases with the fracture's development, and the damage's growth presents nonlinear characteristics. The equation is as follows:

9 $$D = \sum {\frac{{\Delta \varepsilon^{p} }}{{\Delta \varepsilon_{f}^{p} }} = \sum {\frac{{\Delta \varepsilon^{p} }}{{D_{1} (P^{*} + T^{*} )^{{D_{2} }} }}} }$$

where $\Delta \varepsilon^{p}$ is the equivalent plastic strain rate;$\Delta \varepsilon_{f}^{p}$ is the equivalent plastic strain at failure; *D*_1_ and *D*_2_ are damage factors, which is determined by the following method.

10 $$\varepsilon_{p}^{f} = D_{1} (P^{*} + T^{*} )^{{D_{2} }}$$

where $\varepsilon_{p}^{f}$ is the plastic strain to fracture under a constant pressure. According to formula (10), the curve of the pressure dependent fracture strain following the normalized pressure can be determined by test, and the damage factors *D*1 and *D*2 can be determined by the curve^21^.

### The equation of state of pressure

**Figure 8:**
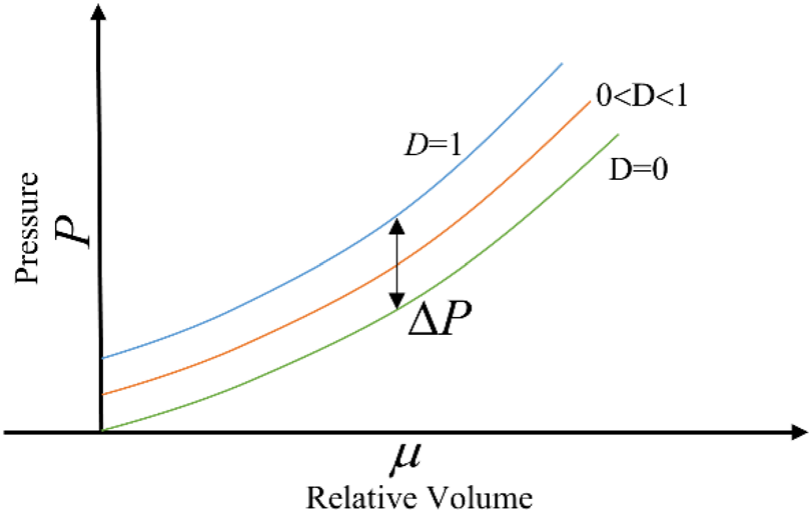
EOS model of the JH-2 constitutive model.

The pressure state equation curve of the JH-2 continuum model is shown in Fig. 8. The pressure state equation reflects hydrostatic pressure and volumetric strain, including pure elastic and plastic failure stages. The equation is as follows:

11 $$\left\{ {\begin{array}{*{20}l} {P = K_{1} \mu + K_{2} \mu^{2} + K_{3} \mu^{3} } \hfill \\ {P = K_{1} \mu + K_{2} \mu^{2} + K_{3} \mu^{3} +\Delta P} \hfill \\ {P = K_{1} \mu } \hfill \\ \end{array} } \right.\begin{array}{*{20}c} {(D = 0,\mu { > }0)} \\ {(0{ < }D \le 1,\mu { > }0)} \\ {(\mu { < }0)} \\ \end{array}$$

where *K*_1_, *K*_2_, and *K*_3_ are material constants, *μ* can be calculated as follows:

12 $$\mu = \frac{\rho }{{\rho_{0} }} - 1$$

where $\rho_{0}$ is the initial density of the material; *ρ* is the current density.

Considering bulking energy, the pressure increment Δ*P* is taken into account in the state equation. When the material is damaged, the strength of the material will weaken, and the elastic energy of the material will also decrease. The reduced elastic energy will be converted into potential energy ^20^, by increasing the pressure increment Δ*P*. The equation of Δ*P* is as follows:

13 $$\Delta P_{{t +\Delta t}} = - K_{1} \mu_{{t +\Delta t}} + \sqrt {\left( {K_{1} \mu_{{t +\Delta t}} +\Delta P_{t} } \right)^{2} + 2\beta K_{1}\Delta U}$$

where Δ*U* is deviator elastic energy; *β* is the part of the incremental elastic energy that is converted into potential energy (0 ≤ *β* ≤ 1).

### Relationship between surrounding rock damage and sonic wave velocity

The relation equation between the rock damage variable and sonic wave velocity before and after rock damage can be obtained:^23^

14 $$D = 1 - \left( {\frac{{\overline{{V_{p} }} }}{{V_{p} }}} \right)^{2}$$

where $\overline{{V_{p} }}$ is the sonic wave velocity of rock mass subjected to blasting load; $V_{p}$ is the sonic wave velocity of rock; *D* is the damage variable of rock after blasting.

The calculation formula of the change rate of sonic wave velocity is as follows:

15 $$k_{v} = 1 - \frac{{\overline{{V_{p} }} }}{{V_{p} }}$$

Criteria for judging the damage of surrounding rock under blasting load by sonic wave test^23^: when the change rate of sonic wave velocity is not more than 10%, the damage of surrounding rock is minimal. When the change rate of sonic wave velocity is more than 10% but less than 15%, the surrounding rock is considered to be slightly damaged by blasting. When the change rate of sonic wave velocity is greater than 15%, the surrounding rock is considered to be damaged by blasting. When the change rate of sonic wave velocity is 10%, the rock damage variable is 0.19. When the change rate of sonic wave velocity is 15%, the rock damage variable is 0.28.

## Simulation of cumulative damage of surrounding rock in cyclic blasting

**Figure 9:**
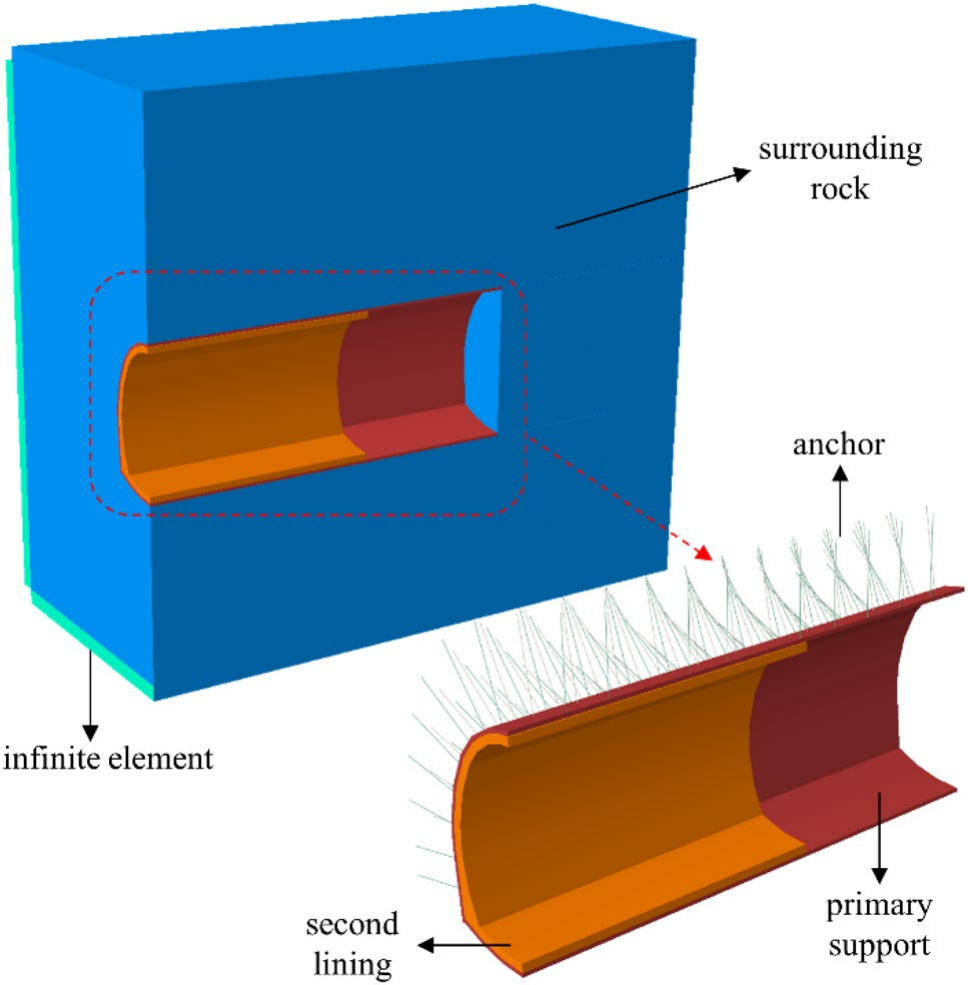
Schematic of numerical model.

Taking the step-blasting construction in the Jinjing tunnel as an example, the numerical model is established. The model size is 40 m × 37 m × 37 m, and the C3D8R element is used for the rock and lining. The beam element is used for the anchor. The data transfer method between solvers in ABAQUS software is used to realize the tunnel cyclic blasting excavation construction. During the simulation of cyclic blasting, the blasting load is applied to the excavation boundary. Before beginning the blasting operation, the rock elements of the cyclic blasting excavation are deleted. The next cyclic blasting is calculated based on all the calculation results of the previous cyclic blasting. The schematic diagram of the numerical model is shown in Fig. 9.

### Material parameters

Since the blasting mainly causes vibration to the tunnel's anchors, primary lining, and secondary lining, damage cannot be considered. Only the material elasticity parameters are considered, as shown in Table 1.

**Table 1:** The physical parameters of anchor, primary lining, and secondary lining.

Materials	Density (kg m^−3^)	Elastic modulus (Gpa)	Poisson’s ratio
anchor	7800	210	0.2
Primary lining	2200	26	0.21
Secondary lining	2500	29.5	0.21

The JH-2 model is used for the rock mass, and its specific parameters are shown in Table 2 according to the literature^20^ and engineering geological survey data.

**Table 2:** The material parameters of the rock materials.

*ρ* (kg m^−3^)	*G* (Gpa)	A	B	n	C	m	$\dot{\varepsilon }_{0}$	*β*	*T* (MPa)
2550	17.8	1.01	0.68	0.83	0.005	0.83	1.0	1.0	45
$\varepsilon_{f\min }^{p}$	$\varepsilon_{f\max }^{p}$	$P_{HEL}$(GPa)	$D_{1}$	$D_{2}$	$K_{1}$(GPa)	$K_{2}$(GPa)	$K_{3}$(GPa)	HEL (GPa)	
0.001	1.0	2.6	0.005	0.7	19.5	− 23	2980	4.5	

### Blast load

**Figure 10:**
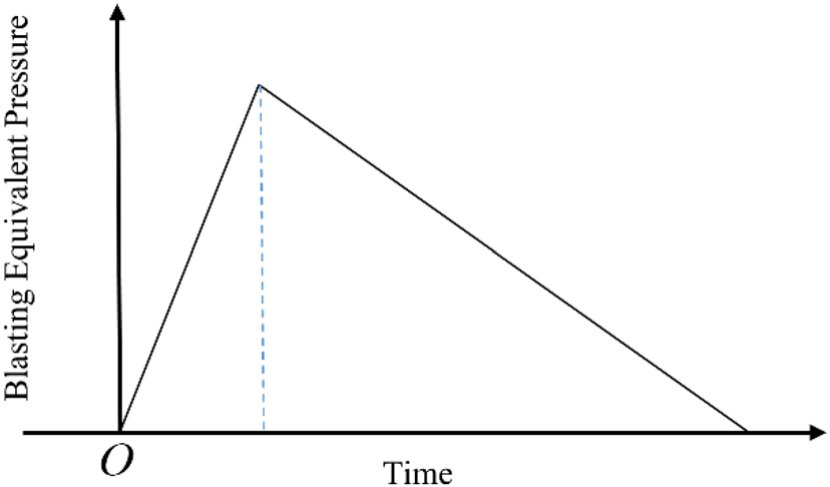
Curve of blasting load.

The calculation cost can be greatly decreased by applying the equivalent blasting load on the elastic boundary instead of modeling the explosion and the gun hole. The triangular equivalent blast load will be adopted in this paper. The blast load curve is shown in Fig. 10.

Charge amount and hole arrangement determine the value of blasting load, and footage determines the range of blasting load. As the influencing factors of blasting load, they affect the damage of the surrounding rock.

The initial detonation pressure on the hole wall can be calculated for uncoupled charges by the following formula^24^.

16 $$P_{0} = \frac{{\rho_{e} D^{2} }}{2(\gamma + 1)}\left( {\frac{{d_{c} }}{{d_{b} }}} \right)^{2\gamma }$$

where *P*_0_ is the initial detonation pressure;$\rho_{e}$ is explosive density; *D* is the detonation velocity of the explosive; *γ* is the isentropic index of explosives, taking the value of 3.0; *d*_c_ is charge diameter; *d*_b_ is the diameter of the hole.For the detonation of a group of holes, the equivalent blast load applied on the elastic boundary can be calculated as follows^21^:

17 $$P_{1} = kP_{0} \left( {\frac{{r_{0} }}{{r_{1} }}} \right)^{{2 + \frac{\mu }{1 - \mu }}} \left( {\frac{{r_{1} }}{{r_{2} }}} \right)^{{2 - \frac{\mu }{1 - \mu }}}$$

where *k* is the influence coefficient of cut hole initiation, which is taken as 10; $r_{0}$ is the radius of the hole; $r_{1}$ is the radius of the crushing zone; $r_{2}$ is the radius of crushing area; *μ *Poisson’s ratio of surrounding rock.

2# emulsion explosive is used in the building of the Jinjing tunnel blast, its density is 1100 kg/m^3^, and detonation velocity is 4000 m/s; according to the relevant engineering data, the equivalent blasting load *P*_1_ at the elastic boundary is 200 Mpa, as calculated by Eq. (17).

### Cumulative damage characteristics of the surrounding rock

A single blasting operation will damage a certain range of adjacent rocks in this excavation area. As the frequency of blasting operations rises, the damage to the surrounding rocks in the cyclic blasting construction section will accrue based on the damage produced by each individual blasting operation, exhibiting the traits of overall surrounding rock damage. The damage caused by blasting in this section will be exacerbated by subsequent blasting. This section analyzes and discusses the cumulative damage characteristics of the single blast building section and the surrounding rock of the cyclic blast.

**Figure 11:**
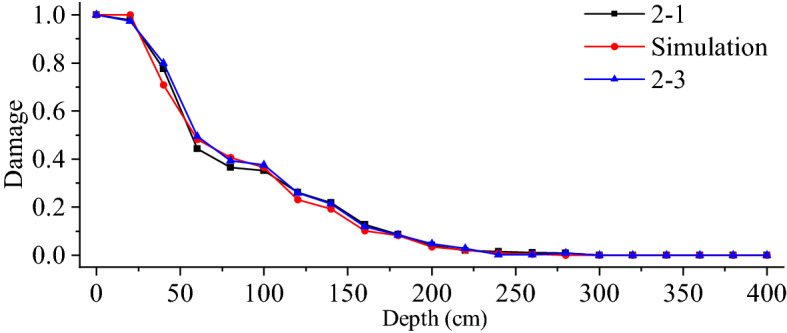
Comparison of field test and numerical simulation results.

The sonic wave velocity of the reserved surrounding rock obtained from the sonic test is substituted into Eq. (14) to get the damage variable in the field, which is compared with the damage variable in the simulation, as shown in Fig. 11. The damage vaule of numerical simulation is extracted by the post-processing function of ABAQUS software. It can be seen from Fig. 11 that the simulation value is consistent with the field measurement value. When the damage variable is 0.19, the damage depths in numerical simulation, field test 1, and field test 2 are 130 cm, 143 cm, and 144 cm, respectively. When the damage variable is 0.28, the damage depths in numerical simulation, field test 1, and field test 2 are 110 cm, 117 cm, and 115 cm, respectively. The average depth deviation for the two damage variable groups is 13.5 cm and 6 cm, respectively. Clearly, the numerical simulation method utilized in this research has a degree of trustworthiness.

#### Cumulative damage characteristics of the overall surrounding rock

**Figure 12:**
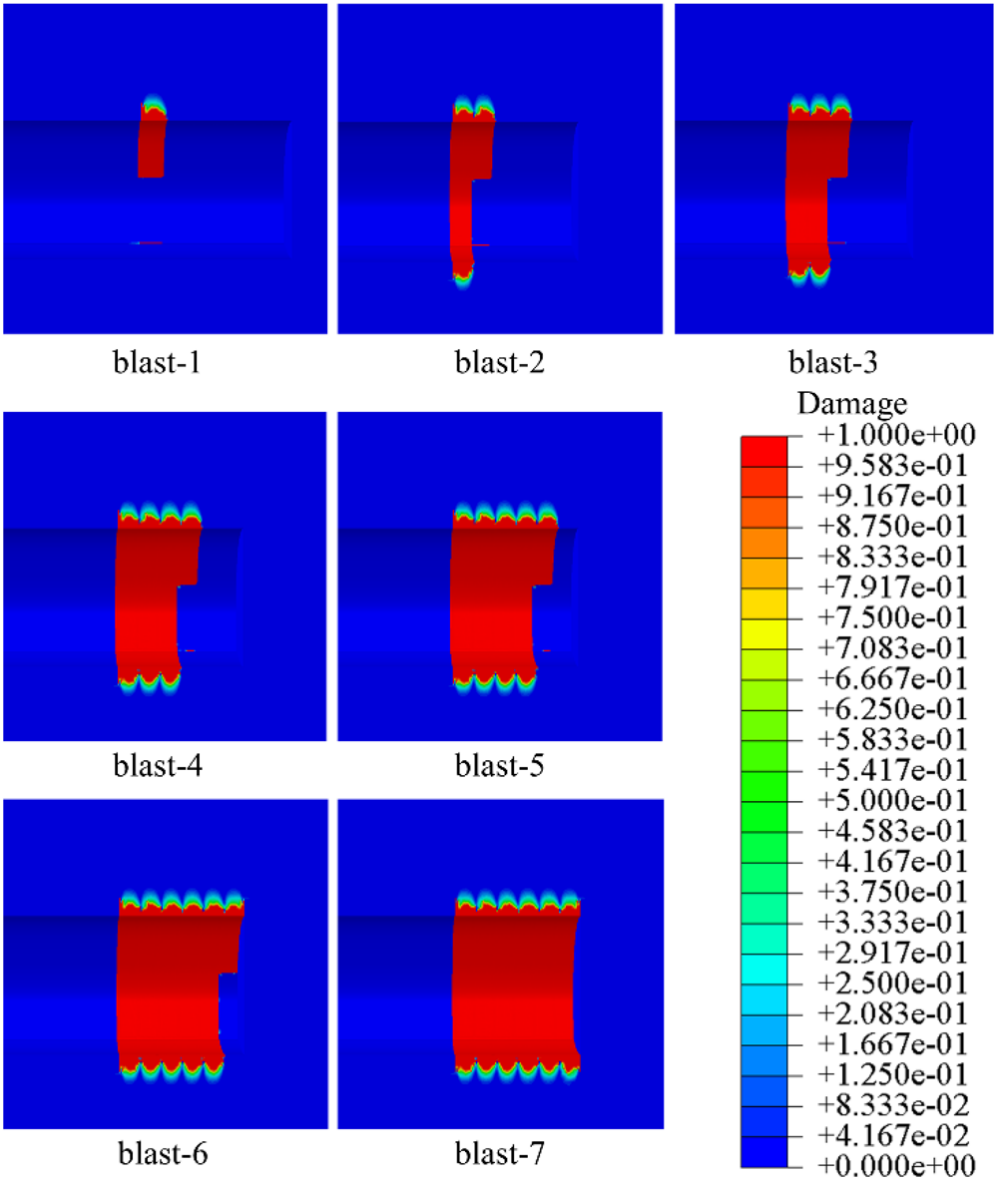
Damage variables of the overall reserved surrounding rock along the tunnel longitudinal.

Figure 12 depicts the distribution of damage factors along the longitudinal direction of the tunnel during the seven excavation cycles of blasting construction. Figure 12 is the damage cloud image of JH-2 model, which was obtained by the post-processing function.

**Figure 13:**
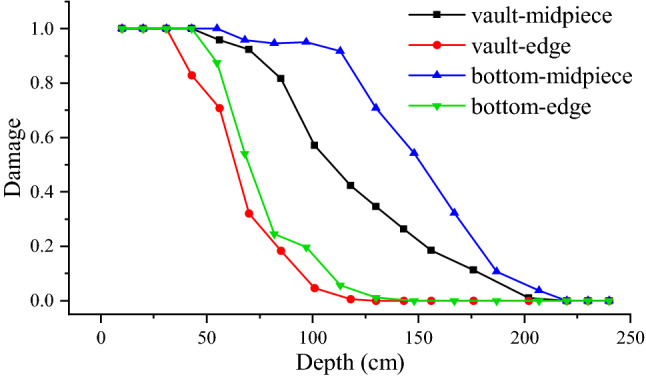
The curve between the damage variable at the edge and center of the periodic damage area and the depth.

In accordance with Fig. 12, along the tunnel's longitudinal axis, the damage distribution of the reserved overall surrounding rock is uniform and symmetrical, exhibiting periodic damage features. The single blasting construction range is the main damage area in each damage period. The length of the periodic damage area is 160 cm, which is slightly longer than the length of the single blasting excavation footage. The vault's periodic damage depth is 202 cm, and the bottom's periodic damage depth is 217.5 cm. Along the longitudinal direction of the tunnel, the periodic damage area of the reserved surrounding rock exhibits a funnel shape. The damage variable value and damage depth at the hole's ends are less than those in the hole’s center. The surrounding rock’s shallow depth damage variable value is more significant, and the degree of damage is higher. With the increase of depth, the damage variable of surrounding rock gradually decreases. Figure 13 shows the relationship between the damage variables at the edge and center of the periodic damage area and the depth.

According to Fig. 13, when the surrounding rock depth grows, the damage variable demonstrates nonlinear attenuation, and the degree of attenuation increases continually. The damage variable of the vault surrounding rock attenuates faster than the damage variable of the bottom surrounding rock, and the damage degree of the arch bottom surrounding rock is slightly higher than that of the vault. The attenuation rate of the damage variable in the periodic damage margin is significantly greater than that in the middle rock, which correlates to the funnel-shaped damage distribution features. The above guidelines show that charge blasting will have a substantial impact on the surrounding rock in the middle region of the hole.

#### Cumulative damage characteristics of surrounding rock in a single excavation section

**Figure 14:**
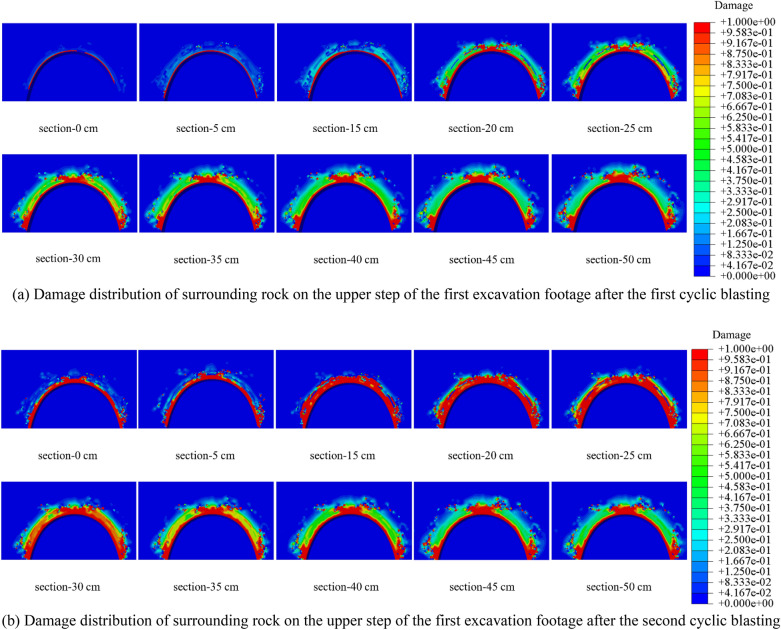
Damage distribution of surrounding rock in different sections of the upper step of the first excavation footage.

**Figure 15:**
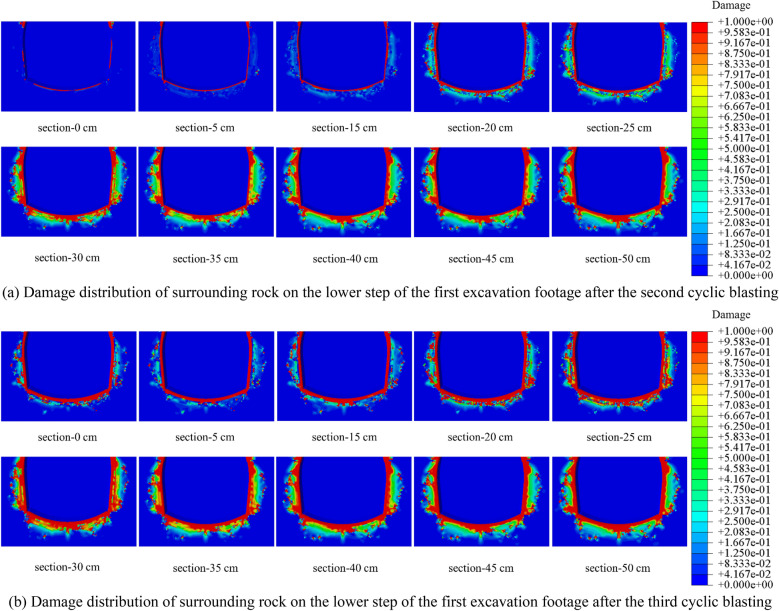
Damage distribution of surrounding rock in different sections of the lower step of the first excavation footage.

To visually observe and analyze the damage in the cross-section of the single excavation and to obtain the cumulative damage characteristics, the view section function in ABAQUS post-processing was used to cut the first excavation. The damage distribution of the cut sections at different distances from the bottom of the hole for the upper and lower steps is shown in Figs. 14 and 15.

**Figure 16:**
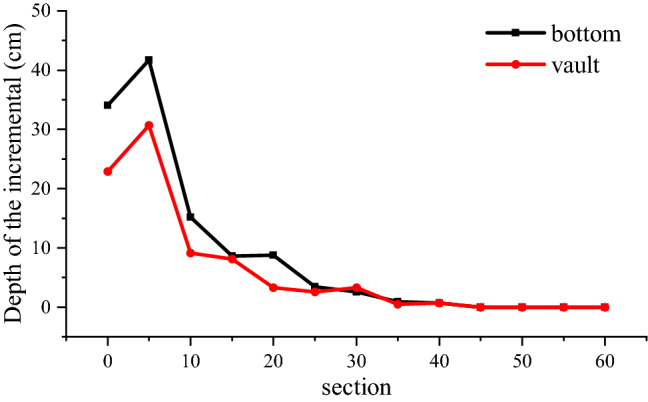
Increment of surrounding rock damage depth in different sections of vault and bottom.

**Figure 17:**
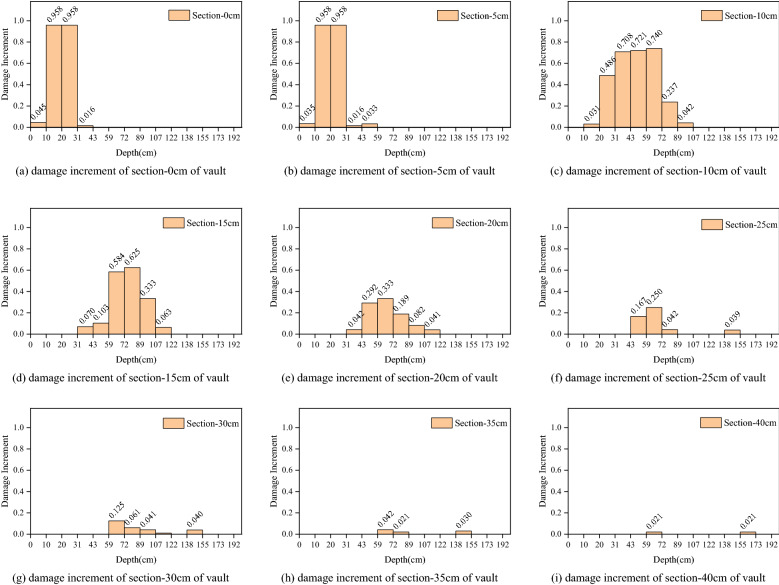
Damage increment distribution along the depth of surrounding rock in different sections of vault.

**Figure 18:**
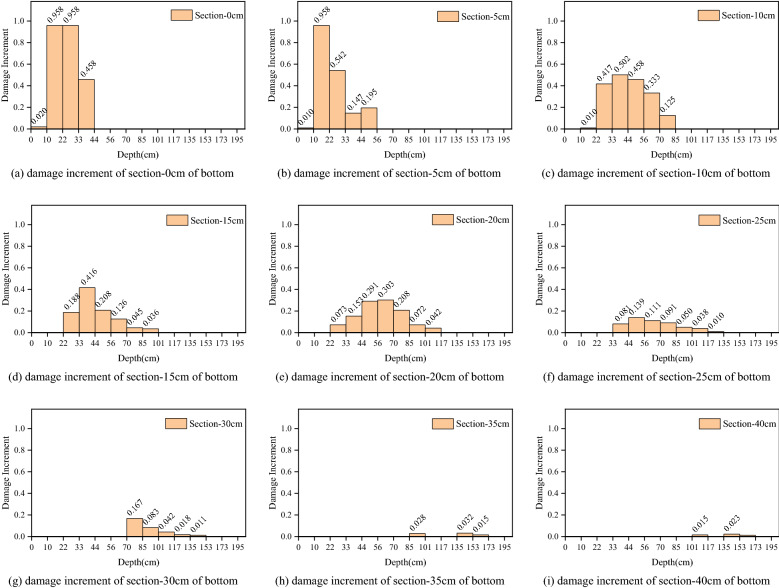
Damage increment distribution along the depth of surrounding rock in different sections of the bottom.

As shown in Figs. 14 and 15, the value and depth of the damage variable will grow as the view section approaches the hole's center, corresponding to the tunnel’s funnel-shaped damage. The blasting force of the latter excavation footage will have an apparent cumulative effect on the damage of the earlier excavation footage’s reserved surrounding rock. The length of the affected area is 40 cm, accounting for 26.7% of the excavation footage. As illustrated in Figs. 16, 17, and 18, the increment of damage depth and damage vale in various sections of the upper step vault and lower step bottom are determined.

According to Fig. 16, the blasting of the latter structure will have a considerable impact on the damage depth of surrounding rock within 15 cm of the bottom of the prior excavation's hole. The upper step's maximum damage depth increase is 30.7 cm, while the lower step's maximum damage depth increment is 41.7 cm. When the distance from the bottom of the hole exceeds 15 cm, the surrounding rock’s damage depth falls significantly. When the distance from the bottom of the hole exceeds 40 cm, the damage depth of the surrounding rock does not rise.

According to Figs. 17 and 18, the damage increment of surrounding rock is greatest within 5 cm of the bottom of the hole, and the maximum increment of the upper and lower steps is 0.958. In the range of 5–40 cm from the bottom of the hole, the cumulative damage increment of surrounding rock reduces consistently, from 0.74 to 0.021 for the upper step and from 0.502 to 0.023 for the lower step. The accumulated damage of surrounding rock will not occur if the distance from the bottom of the hole is greater than 40 cm. The depth range of accumulated damage of surrounding rock within 0–5 cm of the hole's bottom is limited. The upper step's average depth of collected damage is 33.4 cm, and the lower step's average depth of accumulated damage is 40.5 cm. The depth range of accumulated damage within 5–20 cm of the hole’s bottom is vast. The upper step has an average accumulative damage depth of 81.5 cm, while the bottom step has an average accumulative damage depth of 75.6 cm. The depth range of accumulated damage within 20–40 cm of the hole's bottom is limited. The upper step's average accumulative damage depth is 26.8 cm, and the bottom step's average accumulative damage depth is 49.8 cm.

The aforementioned occurrence reflects the three cumulative damage patterns observed in prior excavation footage. The cumulative damage mode of the surrounding rock in the range of 0–5 cm from the bottom of the hole is that the damage increment is greatest at shallow depths. The cumulative damage pattern of the surrounding rock within 5–20 cm of the hole's bottom indicates that the greater damage increment occurs at greater depths. The accumulated damage mode of surrounding rock within 20–40 cm of the hole's bottom is a little damage increment in a shallow depth of surrounding rock.

The damage depth increment and the distribution law of the damage increment of the cross-sectional surrounding rock reveal that the previous excavation's funnel-shaped damage is intensified.

## Conclusion

In this study, the method of sonic wave test and numerical simulation is used to compute and analyze the cumulative damage characteristics of reserved surrounding rock following cyclic blasting. The results can offer pertinent theoretical direction for step blasting tunnel construction's blasting scheme design and lining support design. The following is the conclusion:

1. The cumulative damage value of cyclic blasting based on the JH-2 rock damage model is compatible with the results of the sonic test, and the damage model can more accurately represent the dynamic mechanical behavior of rock materials.
2. The entire reserved surrounding rock in the cyclic blasting excavation section has periodic damage characteristics, and the length of each periodic damage region is 160 cm, 1.07 times the excavation footage, and takes the shape of a funnel along the tunnel's longitudinal direction. The damage depth is 202 cm at the vault and 217.5 cm at the bottom. Each periodic damage region is generated by the combined action of this footage section's blasting construction and the later footage section's blasting construction.
3. The blasting effect of the latter excavation footage construction on the damaged area of the preceding excavation footage's reserved surrounding rock is 40 cm long, accounting for 26.7% of the excavation footage. In the 40 cm influence area, there are three cumulative harm patterns. The surrounding rock has the greatest damage increment at a shallow depth of 0–5 cm from the hole's bottom. At a large depth, the surrounding rock has a greater damage increment between 5 and 20 cm from the bottom of the hole. The surrounding rock shows a slight damage increment at a small depth between 20 and 40 cm from the bottom of the hole. The three cumulative damage patterns show the additional damage law of the surrounding rock in the prior excavation footage caused by the blasting activity in the subsequent excavation footage more clearly. Contrary to common belief, the closer the distance to the latter excavation footage is, the greater the cumulative damage to nearby rock will be. This discovery has implications for tunnel blasting design and tunnel support design.

## Data Availability

The datasets generated during and/or analyzed during the current study are not publicly available but are available from the corresponding author on reasonable request.
